# COGENT: evaluating the consistency of gene co-expression networks

**DOI:** 10.1093/bioinformatics/btaa787

**Published:** 2020-09-15

**Authors:** Lyuba V. Bozhilova, Javier Pardo-Diaz, Gesine Reinert, Charlotte M. Deane

**Affiliations:** Department of Statistics, University of Oxford, Oxford OX1 3LB, United Kingdom; Department of Statistics, University of Oxford, Oxford OX1 3LB, United Kingdom; Department of Statistics, University of Oxford, Oxford OX1 3LB, United Kingdom; Department of Statistics, University of Oxford, Oxford OX1 3LB, United Kingdom

## Abstract

**Summary:**

Gene co-expression networks can be constructed in multiple different ways, both in the use of different measures of co-expression, and in the thresholds applied to the calculated co-expression values, from any given dataset. It is often not clear which co-expression network construction method should be preferred. COGENT provides a set of tools designed to aid the choice of network construction method without the need for any external validation data.

**Availability and implementation:**

https://github.com/lbozhilova/COGENT.

**Supplementary information:**

Supplementary information is available at *Bioinformatics* online.

## 1 Introduction

Gene expression data are a powerful resource for understanding genetic function under different conditions. A common way of exploring this data is through gene co-expression networks (Lee *et al.*, 2004). In these networks, genes are represented by nodes and highly co-expressed gene pairs are connected by edges. Such networks have been used in many ways, including for gene function prediction and the identification of disease- or tissue-relevant gene modules (van Dam *et al.*, 2017).

Gene expression data typically take the form of a matrix, in which rows correspond to genes and columns correspond to samples. Network construction commonly consists of three steps—the data are pre-processed, a measure of co-expression is calculated for every pair of genes and a score cut-off is applied. Different approaches to data pre-processing and normalization exist (Abbas-Aghababazadeh *et al.*, 2018; Park *et al.*, 2003). Further, after normalization co-expression can be calculated in a number of different ways—e.g. via a correlation coefficient or mutual information. A score cut-off is then usually imposed in order to identify gene pairs which are highly co-expressed. Alternatively, weighted networks can be analysed, in which edge weights correspond to levels of co-expression (Langfelder and Horvath, 2008).

There are many available methods for network construction which can be applied to the same dataset, and which then lead to different networks. Enrichment analysis or comparison to orthogonal data, such as protein interaction data, is commonly used for network selection and validation. However, for many species and datasets poor or non-existent functional annotation makes this type of validation difficult. It is therefore often not clear which of the available network construction methods should be prioritized (De Smet and Marchal, 2010).

Here, we introduce COGENT (COnsistency of Gene Expression NeTworks), an R package designed to aid the choice of a network construction pipeline without the need for annotation or external data. COGENT can be used to choose between competing co-expression measures, as well as to inform score cut-off choice. While designed for gene expression data, COGENT can be applied to other cases where network construction relies on similarity profiling, e.g. microbiome or synthetic lethality data.

## 2 Software description

### 2.1 Method overview

Consistent co-occurrence of two gene products in the cell points towards a functional relationship between the genes. Gene product abundance, as well as co-occurrence, is a continuous-time phenomenon, which is experimentally observed at discrete time points or samples. The construction of a gene co-expression network can therefore be thought of as an estimation problem—we aim to infer general co-expression patterns from a limited set of data points. One way of investigating the success of such a procedure is through resampling. Networks constructed from a subset of all available samples will be noisier than the network constructed from the full dataset. However, they should still resemble each other: if subsetting the data results in networks with little to no overlap, then the network construction procedure may be too sensitive to noise in the data.

**Fig. 1.**
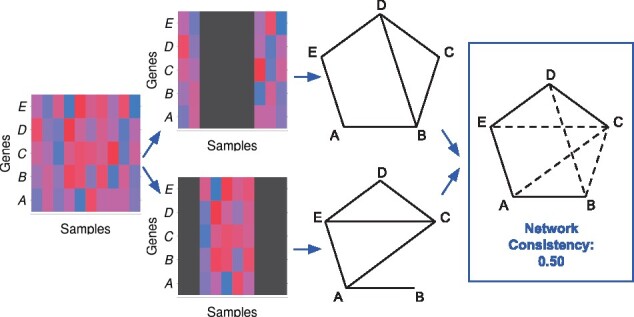
COGENT workflow schematic. In this example, the input data are a gene expression matrix with rows corresponding to genes—in this case A,B,C,D,E—and columns corresponding to samples (far left). First, the expression matrix columns are randomly split into two possibly overlapping groups of equal size (left). Then, a network is constructed from each of the sample groups (right). Finally, the two resulting networks are compared and the consistency between them is calculated (far right). In this example, the two networks have six edges each, and overlap at four of these edges. One measure of their consistency is the Jaccard index between their edge sets (see Supplementary Section S4), which in this case is 0.50

COGENT evaluates network construction methods through iterative resampling. At each step, the gene expression samples are split into two possibly overlapping sets of equal size. The same network construction function f(·) is applied to both sets in order to obtain two gene co-expression networks $G_{1}=(V,E_{1})$ and $G_{2}=(V,E_{2})$. COGENT then calculates several measures of consistency between these two networks. An example workflow for calculating consistency is shown in Figure 1; see Supplementary Section S2 for workflow details and Supplementary Section S4 for different consistency measures.

The entire procedure is repeated multiple times in order to obtain robust results. Network construction methods which result in highly similar pairs of networks $G_{1}\approx G_{2}$ are considered to be consistent. When two or more competing methods are considered, the method exhibiting higher internal consistency should be preferred.

### 2.2 Edge set consistency

Consistency in COGENT is measured through a network comparison step at each iteration. The two networks *G*_1_ and *G*_2_ are considered to be similar if their edge sets are similar ($E_{1}\approx E_{2}$). We measure agreement between *E*_1_ and *E*_2_ using a (weighted) Jaccard index to produce two measures of edge set consistency—global and local similarity (Kao and Porter, 2018; see Supplementary Section S4.1).

Since global similarity scales with network density, a density adjustment is required when methods resulting in different network densities are compared. This is particularly important when choosing a score cut-off. Density adjustment in COGENT is carried out through comparison to random networks generated using a configuration model (see Supplementary Section S4.2).

### 2.3 Node metric consistency

Density-independent network comparison can also be performed by calculating a node metric such as degree or betweenness for all nodes in each of the two networks, and then comparing the obtained metric values. If the aim of downstream network analysis is e.g. to identify genes with many co-expression partners, the degree is a natural node metric to use.

At each COGENT iteration, a node metric set by the user can be applied to *G*_1_ and *G*_2_, resulting in two node metric vectors *d*_1_ and *d*_2_, respectively. These two can be compared in three different ways: via a correlation coefficient, rank *k*-similarity (Bozhilova *et al.*, 2019; Trajanovski *et al.*, 2013) and Euclidean distance; see Supplementary Section S4.3 for details.

## 3 Application

Global and local similarity and adjusted edge consistency, as well as node metric comparisons can all be used to evaluate network consistency. By iteratively resampling the data and measuring network consistency, COGENT can be used to prioritize different network construction pipelines, as well as to inform co-expression cut-offs without the need for external validation data. A full worked example can be found in Supplementary Section S5 and in the COGENT tutorial. COGENT has also been used to assess signed distance correlation as a measure of gene co-expression (Pardo-Diaz *et al.*, 2021). This application further shows that network construction methods prioritized by COGENT also capture more protein–protein interaction data than methods which were not prioritized.

While originally developed for gene expression data, COGENT can also be used for the inspection of other data types. For example, it can be applied to microbiome data in order to identify symbiotic organisms, or to synthetic lethality data in order to identify genetic interactions with high confidence.

## Funding

This work was supported by the EPSRC [EP/L016044/1 to L.V.B. and C.M.D., EP/R512333/1 to J.P.D., G.R. and C.M.D.], the BBSRC [BB/T001801/1 to G.R.], COST [CA15109 to G.R.] and e-Therapeutics plc.

*Conflict of Interest*: none declared.

## Supplementary Material

btaa787_Supplementary_Data
